# Developing Computational Biology

**DOI:** 10.1371/journal.pcbi.0030157

**Published:** 2007-09-28

**Authors:** Philip E Bourne, Steven E Brenner

Scientific research is an international endeavor, and computational biology is no exception. Last year we were fortunate enough to attend conferences and visit laboratories in a number of different countries and developing economies. Some of those countries had well-established programs in computational biology, while others had fledgling efforts in place and big plans. Because of its cost structure, computational biology is a particularly promising field for emerging economies. Each country we visited had unique features in areas such as the education programs they offered, the types of research being undertaken, and the ways that research is funded. Each nation also has unique challenges, and many nations are developing so quickly that people who leave them for study abroad may find programs entirely different when they return.

Given these differences and rapid developments, we feel that there is much we can learn from each other. To this end we have begun a series of *Perspective* articles from computational biologists in a variety of countries, each of whom offers their personal perspectives on the history, current status, and future of computational biology in their region. We asked the authors to describe the specific challenges they have faced, their perceived strengths, as well as to discuss the institutions (government and private), opportunities, and difficulties of computational biology in their country as a whole.

This month we begin the series with a *Perspective* on the development of computational and genomic biology in Mexico from two of the leaders in the field. Dr. Rafael Palacios de la Lama is a foreign member of the US National Academy of Sciences and one of the world's experts on the genetics of *Rhizobium* and genome dynamics. Dr. Julio Collado-Vides is currently a professor and the Director of the Center for Genomic Sciences at the Universidad Nacional Autonoma de México, a leader in understanding regulation of the complete Escherichia coli genome. In their article, we learn about the pioneering effort of a Mexican research group participating in the E. coli genome project. E. coli was the first among still few bacterial genomes with quality predictions of operons and upstream regulatory elements. Various other ambitious projects are under way in Mexico, such as determining the DNA sequence of the genome of Phaseolus vulgaris. To these ends, the authors educate an elite set of undergraduates in genomic sciences.

Beyond Mexico, in the coming months we will be touring a number of countries where computational biology is expanding.

The Intelligent Systems in Molecular Biology (ISMB) conference brought close to a thousand computational biologists to Fortaleza, Brazil, in the summer of 2006. Memories of that experience will revive as we read about the character and nature of research efforts that are being undertaken in Brazil.

We will learn also about the unique challenges of doing computational biology in Cuba, a country impacted by the US embargo, yet with a determined education system and a strong research emphasis. The passion of Cuban scientists is an inspiration, and their soup-to-nuts approach to research represents an interesting solution to their situation.

We can also expect perspectives from South Africa, Thailand, Argentina, and China, shedding light on current approaches in these countries to a young, rapidly evolving, and changing discipline.

We hope reading these accounts will inspire you to comment, or to write a *Perspective* on computational biology in your part of the world. If you are interested, please e-mail us at ploscompbiol@plos.org, and we will be happy to send you guidelines for a submission.

The pursuit of scientific endeavors around the world allows individuals and nations to capitalize on their potential. It furthers progress and mutual understanding where political and other means have reached their limits. We hope you will learn from the differing approaches and enjoy reading these viewpoints as much as we did. 

